# Higher proportions of circulating CXCR3+ CCR6− Tfh cells as a hallmark of impaired CD4+ T-cell recovery in HIV-1-infected immunological non-responders

**DOI:** 10.1128/mbio.00575-25

**Published:** 2025-03-25

**Authors:** Ruthu Nagaraju, Pruthvi S. Gowda, Durai M. Gunasekaran, Anita S. Desai, Udaykumar Ranga, Ramesh N. R. Masthi, Manjunatha M. Venkataswamy

**Affiliations:** 1 Department of Neurovirology, National Institute of Mental Health and Neurosciences 683889 , Bengaluru, Karnataka, India; 2 Department of Community Medicine, Kempegowda Institute of Medical Sciences Hospital & Research Centre, Bengaluru, Karnataka, India; 3 Department of Biostatistics, National Institute of Mental Health and Neurosciences 29148 , Bengaluru, Karnataka, India; 4 Jawaharlal Nehru Centre for Advanced Scientific Research 29130 , Bengaluru, Karnataka, India; The Ohio State University, Columbus, Ohio, USA

**Keywords:** HIV-1, immunological non-responders, altered CD4+ T-cell subsets, impaired immunological reconstitution, circulatory T follicular helper cells, cTfh, CXCR3+ CCR6− cTfh cells

## Abstract

Despite long-term suppressive antiretroviral therapy (ART), immune dysregulation due to impaired reconstitution of CD4+ T cells is a major hurdle for reducing morbidity and mortality in HIV-1-infected immunological non-responders (INRs, CD4+ T cells ≤350 cells/µL). To evaluate potential immunological factors associated with impaired CD4+ T-cell reconstitution, we performed comprehensive immunophenotyping of multiple subsets of CD4+ T cells among HIV-1-infected individuals with high (>350 cells/µL) and low (≤350 cells/µL) CD4+ T cells, either ART-naïve or ART-exposed (median, 10 years). In comparison to other groups, INRs showed exclusively elevated proportions of CXCR3+ CCR6− Th1-like circulatory T follicular helper (cTfh1) CD4+ T cells, correlating negatively with CD4+ T cells (*r* = −0.6769, *P* < 0.0001), suggesting a strong association with incomplete CD4+ T-cell recovery. In contrast, compared to INRs, higher proportions of CXCR3− CCR6+ Th17-like cTfh cells (cTfh17) in immunological responders (IRs, CD4+ T cells >350 cells/µL) showed no correlation with CD4+ T-cell counts, suggesting a lack of association with CD4+ T-cell recovery. Additionally, proportions of activated (CD4+ CD38+ HLA-DR+) and regulatory (CD4+ CD25+/hi CD127−/lo) CD4+ T cells were increased in INRs compared to IRs, as previously known. A negative correlation was also observed between the CD4+ T-cell counts and activated (*r* = −0.6726, *P* < 0.0001) or regulatory (*r* = −0.5627, *P* < 0.0001) CD4+ T-cell proportions among IRs and INRs. Our study highlights that immune dysregulation associated with skewing of cTfh cells toward CXCR3+ CCR6− Th1-like phenotype may be the leading cause of inefficient CD4+ T-cell recovery in INRs and can serve as a hallmark of impaired CD4+ T-cell reconstitution.

**IMPORTANCE:**

The altered proportions of CD4+ T-cell subsets in immunological non-responders (INRs) indicate their involvement in poor CD4+ T-cell reconstitution. Reversing these alterations may help prevent the loss of CD4+ T cells. Particularly, blocking cTfh-cell polarization toward CXCR3+ CCR6− cTfh-cell subset may help restore CD4+ T-cell counts in INRs, thereby preventing increased risk of morbidity and mortality.

## INTRODUCTION

CD4+ T cells (abbreviated as CD4+) are gradually depleted in human immunodeficiency virus (HIV)-infected individuals in the absence of antiretroviral therapy (ART) (1, 2). Most of the HIV-infected people recover their CD4+ counts to normal (≥500 cells/µL) with suppressive ART (suppressed viral load: undetectable to <1,000 copies/mL after initiation of ART [3–5]). HIV-1-infected individuals who recover their CD4+ counts to >350 or ≥500 cells/µL are termed immunological responders (IRs) (4, 6). However, despite long-term suppressive ART, about 10%–40% of the HIV-1-infected individuals fail to restore their CD4+ counts and are referred to as immunological non-responders (INRs, whose CD4+ counts remain ≤350 cells/µL). These individuals are at an increased risk of developing AIDS (acquired immunodeficiency syndrome) and non-AIDS-related comorbidities and mortality (1, 7, 8). Poor CD4+ reconstitution is a multifactorial condition with several risk factors such as persistent immune activation and inflammation, lymphoid fibrosis, thymic deficiency, microbial translocation, involvement of auto-reactive anti-CD4+ T-cell antibodies, residual viral replication, lower pre-treatment CD4+ counts and activation-induced apoptosis (6, 9–11). However, specific factors and underlying mechanisms are unclear. Unraveling specific factors that cause impaired immune reconstitution can inform newer therapeutic approaches to restore CD4+ counts, thereby reducing comorbidities among INRs.

We hypothesized that the proportions of crucial subsets of CD4+ would be altered in INRs and correlate with non-recovery of CD4+ counts despite ART. Though there are several reports on alterations among individual subsets of CD4+ among INRs from Western and African countries (9, 12–17), a comprehensive assessment of multiple subsets of CD4+ and their association with impaired CD4+ recovery is lacking. Particularly in light of a recent report on the impact of geographical region on inflammatory markers among HIV-infected individuals on ART (18), understanding the factors responsible for impaired immune reconstitution in HIV-1-infected subjects from India is of significance.

We present a cross-sectional study performed to delineate the alterations in CD4+ subsets before ART initiation and after prolonged ART exposure. To the best of our knowledge, this is the first study to determine alterations among a wide range of CD4+ subsets simultaneously in multiple groups that include long-term treated and untreated HIV-1-infected individuals with low (≤350 cells/µL) and high (>350 cells/µL) CD4+ counts. The peripheral CD4+ subsets assessed were T regulatory (Tregs), resting CD4+, CD69+ (early activation marker) CD4+, CD38+ HLA-DR+ (late activation markers) activated CD4+, cytolytic CD4+, naïve T cells (TN), effector T cells (TEFF), memory subsets like central-transitional memory T cells (TCM-TTM), and effector memory T cells (TEM), T-helper (Th) subsets (Th1, Th2, and Th17), CXCR5+ CD4+ (cTfh cells), helper-like cTfh subsets (cTfh1, cTfh2, and cTfh17), CD31 expressing total CD4+ (CD31+ CD4+), recent thymic emigrants (RTEs), and CD31+ memory subsets.

## RESULTS

Of the total 131 participants recruited, 38 were HIV-1 seronegative healthy controls (HC), 28 each among IRs and INRs, untreated HIV-1-infected individuals with CD4+ counts >350 cells/µL (UT^>350^) were 15, and those with CD4+ counts ≤350 cells/µL (UT^≤350^) were 22. We observed no significant difference in age (mean) between the groups. IRs and INRs had a comparable duration of exposure to ART (median of 10 years and 9.5 years, respectively).

At recruitment, the viral load of all the treated subjects was either <1,000 copies/mL or below the detection level, except for two participants (7.14%) out of 28 in the INR group who had a viral load >1,000 copies/mL (Table 1). These data confirmed suppressed viremia (HIV-1 RNA; below detection level or <20 to <1,000 copies/mL [3–5]) in most study participants on ART despite CD4+ cell-count differences (Table 1). Analysis of socioeconomic status revealed no difference in education or economic status among the treated or untreated groups. A higher number of employed subjects was noticed in INRs and UT^≤350^ than in the IRs and UT^>350^ group (see Table S1 at https://figshare.com/s/4d69d02499e92beeb30d).

**TABLE 1:** Demographic data of study participants at the time of recruitment^*a*^^,^^*d*^

Groups	HC	IRs (CD4 > 350)	INRs (CD4 ≤ 350)	UT^>350^ (CD4 > 350)	UT^≤350^ (CD4 ≤ 350)
No.	38	28	28	15	22
Females	18 (47.36)	15 (53.57)	10 (35.71)	11 (73.33)	8 (36.36)
Males	20 (52.63)	13 (46.43)	18 (64.28)	3 (20)	14 (63.63)
Transgender	0 (0)	0 (0)	0 (0)	1 (6.66)	0 (0)
Age, years^*b*^	40.42 (21–64)	46.14 (36–59)	45.46 (23–60)	37.6 (20–65)	43.9 (29–66)
ART duration, years^*c*^	NAP	10 (8.25–11)	9.5 (5–12)	0	0
CD4+ (cells/µL)	951.3**^*b*^** (362–1,520)	648.5**^*b*^** (360–1,065)	227.8**^*b*^** (43–306)	475^*c*^ (395–539)	191.5^*c*^ (93.75–277.5)
Viral load (copies/mL)					
>1,000	NAP	0 (0)	2 (7.14)	NT***^e^***	NT**^*e*^**
<1,000		4 (14.28)	6 (21.42)		
TND		22 (78.57)	11 (39.28)		
NT		2 (7.1)^*e*^	9 (32.14)**^*e*^**		

^*a*^ Data are presented as no. (%) unless otherwise indicated.

^*b*^ Mean (range).

^*c*^ Median (IQR).

^*d*^ IQR, interquartile range; UT^>350^, untreated HIV-1-infected group with CD4+ counts >350 cells/µL; UT^≤350^, untreated HIV-1-infected group with CD4+ counts ≤350 cells/µL; NAP, not applicable; NT, not tested; TND, target not detected. Suppressed viremia: HIV-1 RNA not detectable or <20 to <1,000 copies/mL (3–5).

^*e*^ Viral load was TND 6 months prior or later to recruitment.

### Delayed ART impacts CD4+ recovery, irrespective of gender

**Fig 1:**
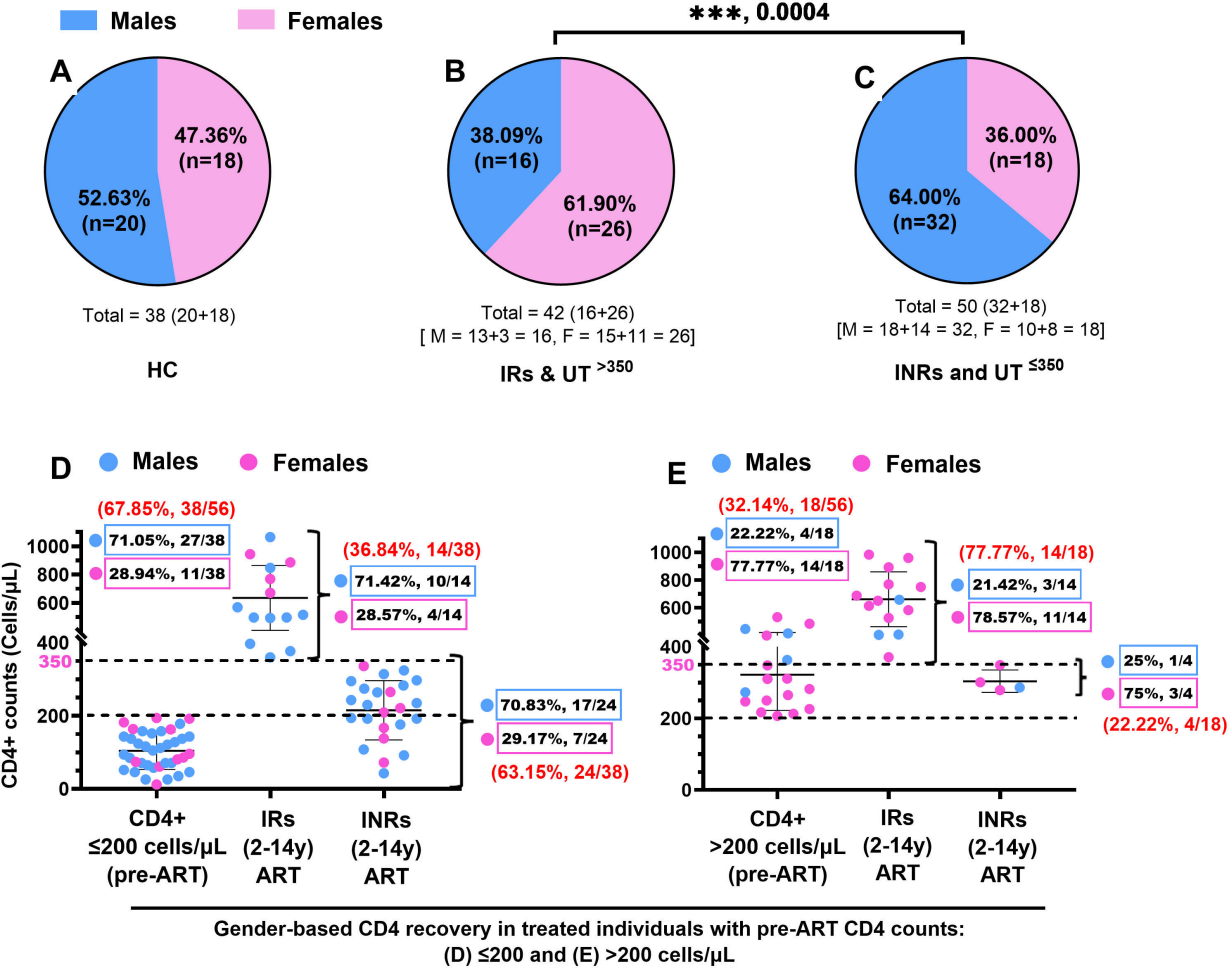
CD4+ non-restoration is prevalent in those with delayed ART. The number of females vs males in the (A) HIV-1-uninfected group (HC), (B) HIV-1-infected groups with high CD4+ (IR and UT^>350^), and (C) HIV-1-infected groups with low CD4+ (INR and UT^≤350^). Gender-based CD4+ recovery after long-term ART in the treated groups (IRs and INRs) with (D) delayed ART (CD4+ counts ≤200 cells/µL) and (E) CD4+ counts >200 cells/µL at ART initiation. Fisher’s test has been used for analyzing categorical data. *P < 0.05, **P < 0.01, ***P < 0.001, ****P < 0.0001, ns: not significant. HC, healthy controls; IRs, HIV-1-infected individuals on ART with CD4 counts >350 cells/µL; INRs, HIV-1-infected individuals on ART with CD4 counts ≤350 cells/µL; UT^>350^, untreated HIV-1-infected individuals with CD4+ counts >350 cells/µL; and UT^≤350^, untreated HIV-1-infected individuals with CD4 counts ≤350 cells/µL.

A higher number of males was observed in the groups with low CD4+ irrespective of ART; INRs (64.28%) and UT^≤350^ (63.63%), compared to the untreated group with high CD4+ (UT^>350^) that comprised a majority of females (73.33%) (Table 1). However, the proportions of males and females were comparable in the IR (Table 1) and HC (Fig. 1A) groups. On combining the groups with high CD4+ (IRs and UT^>350^) and those with low CD4+ (INRs and UT^≤350^), regardless of ART, females were found to be the majority in the high CD4+ groups (61.9%) (Fig. 1B). In comparison, males predominated in the low CD4+ groups (64.0%) (Fig. 1C). The differences in the distribution of male and female subjects in the high and low CD4+ groups were statistically significant (*P* = 0.0004) (Fig. 1B and C).

Furthermore, previous studies have reported that delayed ART (i.e., ART initiation at CD4+ counts ≤200 cells/µL) leads to poor CD4 recovery despite long-term ART (7, 19, 20). To determine whether CD4+ recovery is dependent on gender and/or delayed ART, data of CD4+ counts pre-ART were obtained from the medical records of the long-term ART-treated individuals (IRs and INRs combined) and segregated the treated subjects (*N* = 56) as those with CD4+ counts ≤200 cells/µL (*n* = 38) and >200 cells/µL (*n* = 18) (Fig. 1D and E). Among the 56 treated individuals, a majority (67.85%, 38/56) had delayed ART, and the rest (32.14%, 18/56) had pre-ART CD4+ counts >200 cells/µL (Fig. 1D and E). Among the 38 individuals with delayed ART, 36.84% (14/38) were IRs and 63.15% (24/38) were INRs (Fig. 1D). The IRs included 71.42% (10/14) males and 28.57% (4/14) females. Likewise, INRs included 70.83% (17/24) males and 29.17% (7/24) females (Fig. 1D).

The group with pre-ART CD4+ counts >200 cells/µL (32.14%, 18/56) had 77.77% (14/18) IRs and 22.22% (4/18) INRs, in contrast to the delayed ART group (Fig. 1E). However, the IRs included 21.42% (3/14) males and 78.57% (11/14) females, while the INRs included 25% (1/4) males and 75% (3/4) females (Fig. 1E). The similar proportions of male and female subjects in IRs and INRs, among both groups with delayed ART or pre-ART CD4+ counts >200 cells/µL, indicate that CD4+ recovery or failure is impacted by delayed ART, independent of gender.

### CD4+ counts negatively correlate with Tregs and activated CD4+ among treated groups

**Fig 2:**
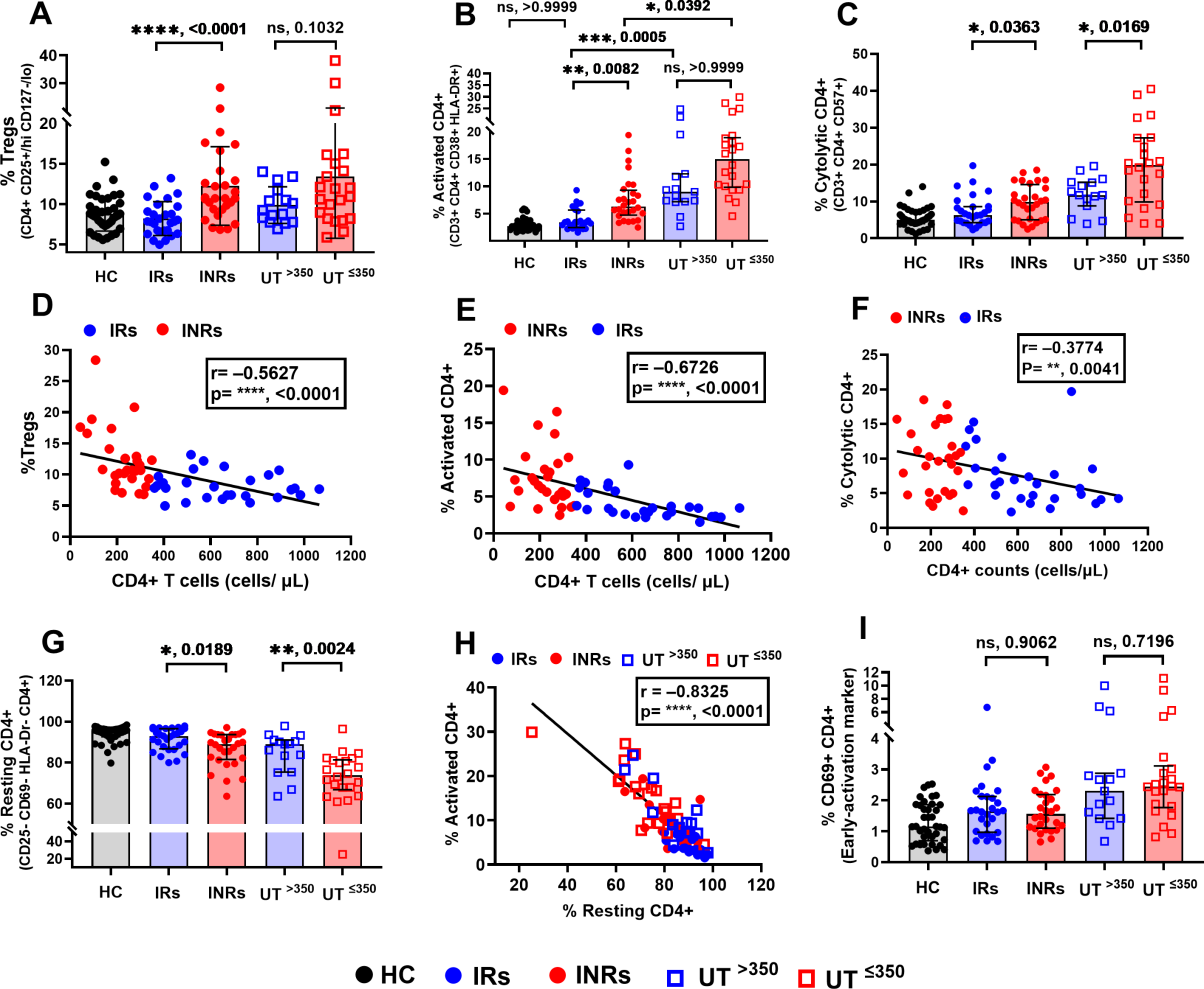
Proportions of Tregs and activated CD4+ associated with low CD4+ counts in INRs. Proportions of (A) Tregs, (B) activated CD4+ (CD38+ HLA-DR+ CD4+), and (C) cytolytic CD4+. Correlation of CD4+ counts with proportions of (D) Tregs, (E) activated CD4+, and (F) cytolytic CD4+ among infected groups. (G) Proportions of resting CD4+ and (I) CD69+ CD4+ between groups. (H) Correlation between frequencies of activated and resting CD4+ among infected groups. Differences between the two groups were analyzed using the Mann-Whitney test (A, C, G, and I). Non-parametric Kruskal-Wallis with Dunn’s multiple comparison test was used for multiple group comparisons (B). Spearman correlation was used to determine the association between variables (D, E, F, and H). *P < 0.05, **P < 0.01, ***P < 0.001, ****P < 0.0001, ns: not significant. Each data point represents a single individual. Black solid circles, HC; blue solid circles, IRs; red solid circles, INRs; blue squares, UT^>350^; and red squares, UT^≤350^.

Although HIV-1-infected groups were classified based on absolute CD4+ counts and the presence or absence of ART, all our subsequent analyses of CD4+ subsets were based on their proportions. Among the treated groups, INRs had significantly higher proportions of Tregs and activated CD4+ (CD38+ HLA-DR+; late activation markers) compared to the IRs (Fig. 2A and B), and both subsets showed a strong negative correlation (*r* = –0.5627, *P* < 0.0001 and *r* = –0.6726, *P* < 0.0001, respectively) with CD4+ counts (Fig. 2D and E). Whereas, between the untreated groups, though statistically not significant, an increasing trend in proportions of Tregs and activated CD4+ was observed in UT^≤350^ compared to UT^>350^ (Fig. 2A). However, correlation analysis revealed a lack of or a weak negative correlation with CD4+ counts for both the subsets, respectively (*r* = –0.2497, *P* = 0.1361 and *r* = –0.3508, *P* = 0.0333) (see Fig. S2 at https://figshare.com/s/4d69d02499e92beeb30d). CD69 is an early activation marker expressed on T cells (21, 22). We found no differences in CD69+ CD4+ proportions between either IRs and INRs or UT^>350^ and UT^≤350^ (Fig. 2I). The assessment of CD57-expressing cytolytic CD4+ revealed that the groups with low CD4+ (INR and UT^≤350^) had significantly higher proportions than the groups with high CD4+ (IR and UT^>350^), regardless of ART (Fig. 2C). Furthermore, they showed a weak negative correlation with CD4+ counts among treated (*r* = –0.3774, *P* = 0.0041) or untreated (*r* = –0.3167, *P* = 0.0562) groups (Fig. 2F; Fig. S2C).

In contrast, proportions of resting CD4+ were significantly lower in groups with low CD4+ (INR and UT^≤350^) compared to that of high CD4+ groups (IR and UT^>350^), treated or not (Fig. 2H). A strong negative correlation between the proportions of activated CD4+ and resting CD4+ (*r* = –0.8325, *P* < 0.0001) was observed (Fig. 2I), indicating an inverse relationship. These results indicate that the proportions of Tregs and activated CD4+ are strongly associated with CD4+ non-restoration and resting CD4+ are associated with CD4+ recovery.

### Central-transitional memory CD4+ predominates in low CD4+ subjects, irrespective of ART

**Fig 3:**
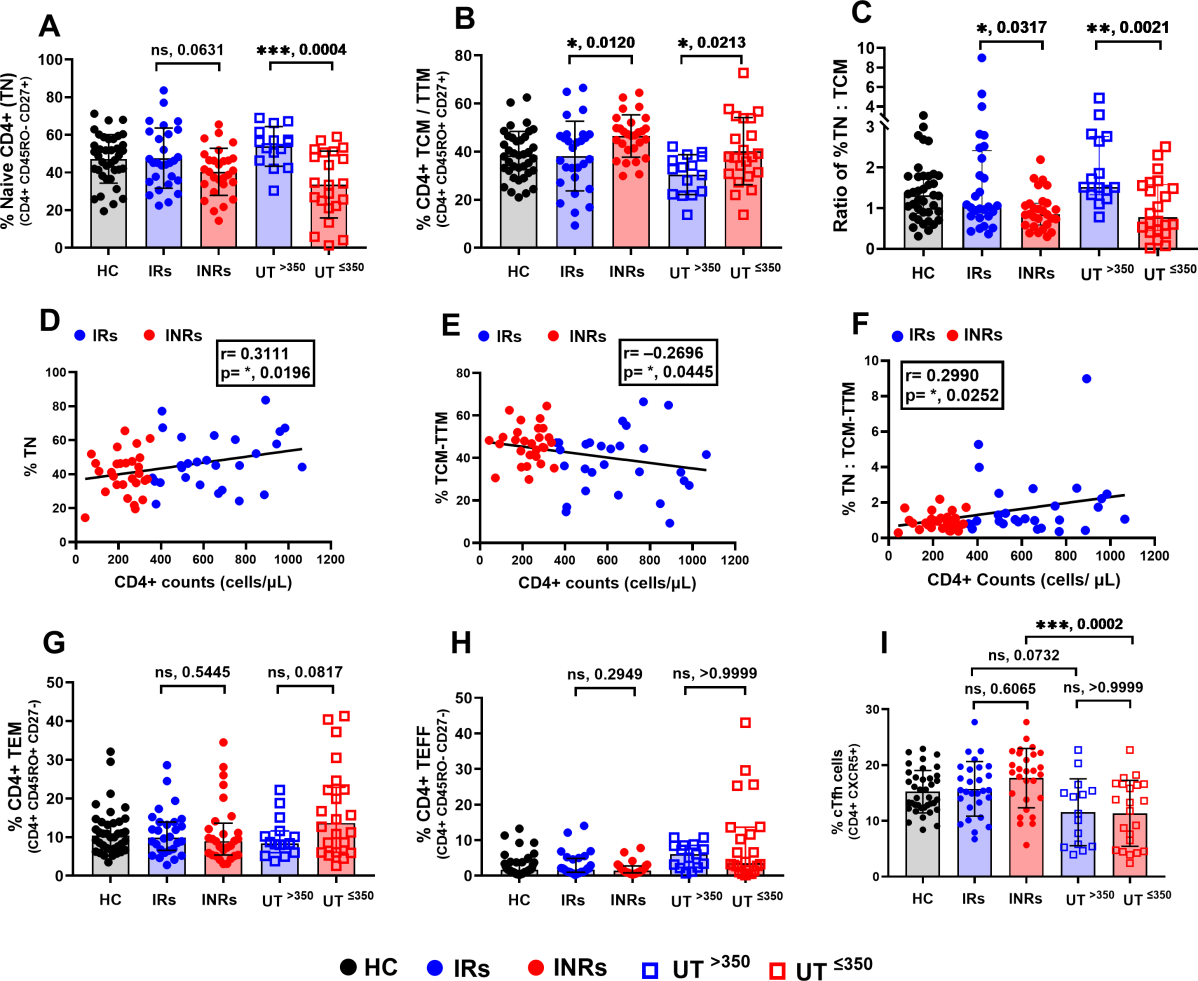
Proportions of central-transitional memory CD4+ (TCM-TTM) predominate in subjects with low CD4+, irrespective of ART. Proportions of (A) naïve (TN), (B) central and transitional memory (TCM-TTM), (C) ratio of % TN:TCM-TTM. Correlation between CD4+ counts and proportions of (D) TN, (E) TCM-TTM, and (F) ratio of TN:TCM-TTM among treated groups. Proportions of (G) effector memory (TEM), (H) effector CD4+ (TEFF), and (I) cTfh cells among the groups. Differences between the two groups were analyzed using the unpaired *t*-test (A and B) or Mann-Whitney test (C, G, and H). One-way ANOVA (analysis of variance) was used for multiple group comparisons (I). Pearson (D and E) or Spearman (F) correlation was used to determine the association between variables. *P < 0.05, **P < 0.01, ***P < 0.001, ****P < 0.0001, ns: not significant. Each data point represents a single individual. Black solid circles, HC; blue solid circles, IRs; red solid circles, INRs; blue squares, UT^>350^; and red squares, UT^≤350^.

We further determined the proportions of other CD4+ subsets, including cTfh, naïve (TN), TCM-TTM, effector memory (TEM), and effector (TEFF) CD4+ T cells. We noted a significantly reduced proportion of TN in the group UT^≤350^ compared to UT^>350^ (Fig. 3A). Among treated groups, a decreasing trend in proportions of TN with no statistical significance was observed in INRs compared to IRs (*P* = 0.0631) (Fig. 3A). Whereas a significantly higher proportion of TCM-TTM and reduced ratio of TN to TCM-TTM were observed in both the low CD4+ groups (INR and UT^≤350^) compared to the high CD4+ groups (IR and UT^>350^), irrespective of ART (Fig. 3B and C). Furthermore, a weak positive correlation between CD4+ counts and TN or ratio of TN:TCM-TTM was noted among both treated (*r* = 0.3111, *P* = 0.0196 or *r* = 0.2990, *P* = 0.0252) (Fig. 3D and F) and untreated groups (*r* = 0.4759, *P* = 0.0029 or *r* = 0.4260, *P* = 0.0086) (see Fig. S2 at https://figshare.com/s/4d69d02499e92beeb30d). Whereas between CD4+ counts and TCM-TTM, a weak negative correlation was observed among both treated (*r* = –0.2696, *P* = 0.0445) and untreated (*r* = –0.4161, *P* = 0.0104) groups (Fig. 3E; Fig. S2E). These results indicate the possible simultaneous depletion of TN and their increased differentiation into TCM-TTM in both the groups with low CD4+ counts, regardless of ART.

Additionally, an increasing trend in the proportions of TEM was noticed in UT^≤350^ than in UT^>350^ (Fig. 3G), with no differences observed in TEFF and cTfh cells between IRs and INRs or UT^>350^ and UT^≤350^, irrespective of CD4+ counts (Fig. 3H and I). These results suggest a lack of association of TEM, TEFF, or cTfH cells with CD4+ recovery in INRs. Interestingly, treated groups showed higher proportions of cTfh cells than untreated groups (*P* = 0.0732 and 0.0002) (Fig. 3I), suggesting their restoration upon ART introduction.

### Thymic dysfunction does not underlie CD4+ non-restoration in INRs

CD31 or platelet endothelial cell adhesion molecule-1 (PECAM-1) is used as a marker of RTEs, a measure of thymic function (1, 23, 24). Higher expression of CD31 on naïve CD4+ (TN) enables quantification of RTEs (CD31+ TN), as it progressively declines with increased cell division and differentiation (i.e., CD31− CD4+) (25–27). We quantified the proportions of CD31 expression on total CD4+ cells and their subtypes (RTEs, memory, and effector cells) to know whether low CD4+ counts were due to inefficient thymic output.

**Fig 4:**
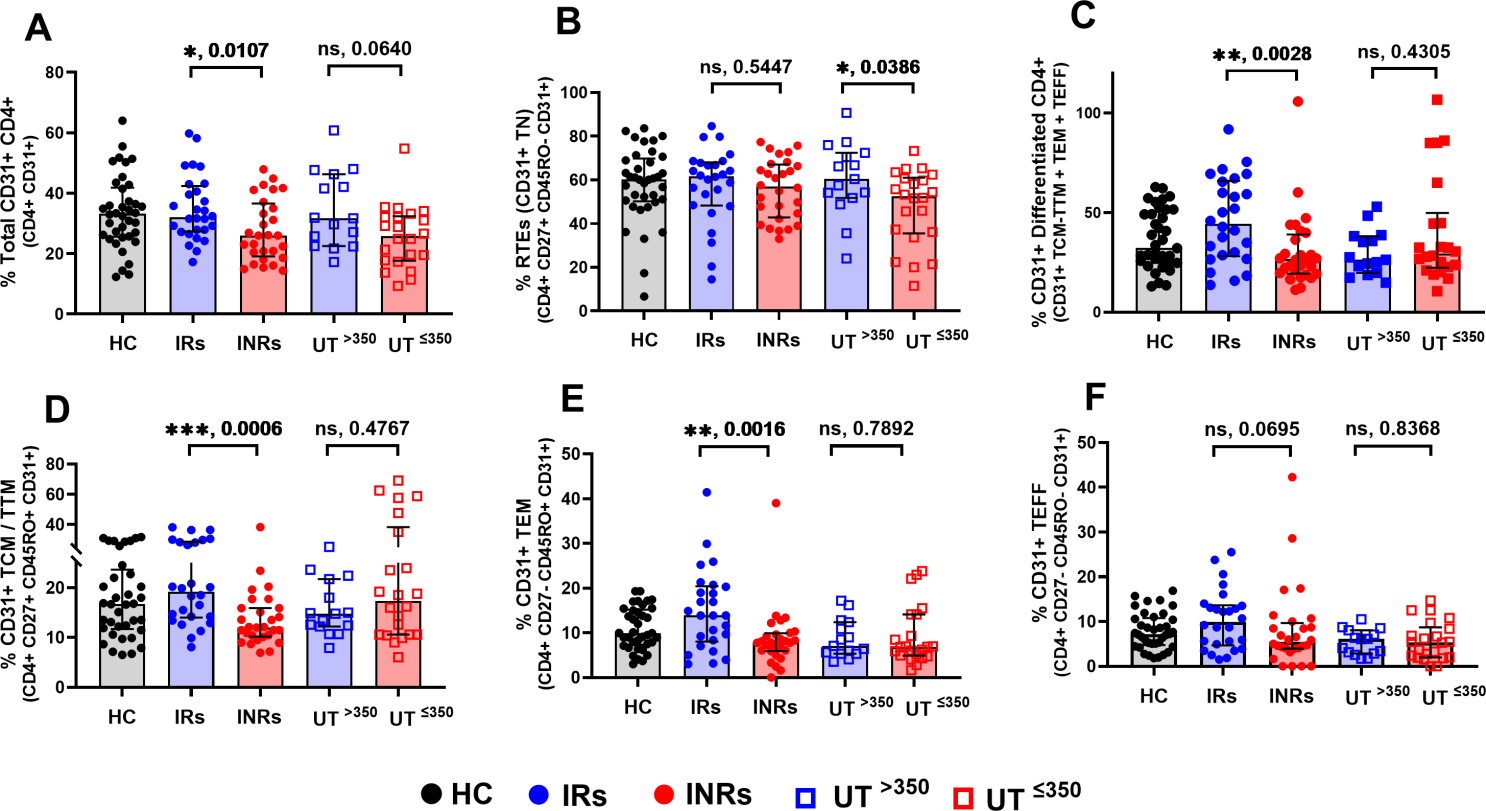
Thymic deficiency in the UT^≤350^, but not in INRs. Proportions of CD31+: (A) total CD4+, (B) RTEs (CD31+ TN), (C) differentiated subsets of CD4+, (D) TCM-TTM, (E) TEM, and (F) TEFF between the groups. Differences between the two groups were analyzed using the Mann-Whitney test. *P < 0.05, **P < 0.01, ***P < 0.001, ****P < 0.0001, ns: not significant. Each data point represents a single individual. Black solid circles, HC; blue solid circles, IRs; red solid circles, INRs; blue squares, UT^>35^; and red squares, UT^≤350^.

We observed lower proportions of total CD31+ CD4+ in INRs compared to IRs and a decreasing trend in UT^≤350^ than in UT^>350^ (Fig. 4A). However, RTEs (CD31+ TN) were significantly lower in UT^≤350^ compared to UT^>350^, with no difference between IRs and INRs (Fig. 4B). In contrast, CD31+ differentiated subsets (TCM-TTM + TEM + TEFF) were significantly lower in INRs compared to IRs, with no differences observed between UT^≤350^ and UT^>350^ (Fig. 4C). Further analysis revealed that the lower CD31+ differentiated subsets in INRs were due to reduced CD31+ memory cells (CD31+ TCM-TTM and CD31+ TEM) (Fig. 4D and E) contributing to lower proportions of total CD31+ CD4+ in INRs (Fig. 4A) and not CD31+ TEFF (Fig. 4F). These results indicate that thymic deficiency partially contributes to reduced CD4+ counts in the UT^≤350^ group, but not in INRs, and reduced CD31+ memory cells suggest an increased differentiation or peripheral proliferation of TN (CD31− CD4+, loss of CD31 expression).

### Elevated CXCR3+ CCR6− cTfh cells strongly correlate with CD4+ non-restoration in INRs

**Fig 5:**
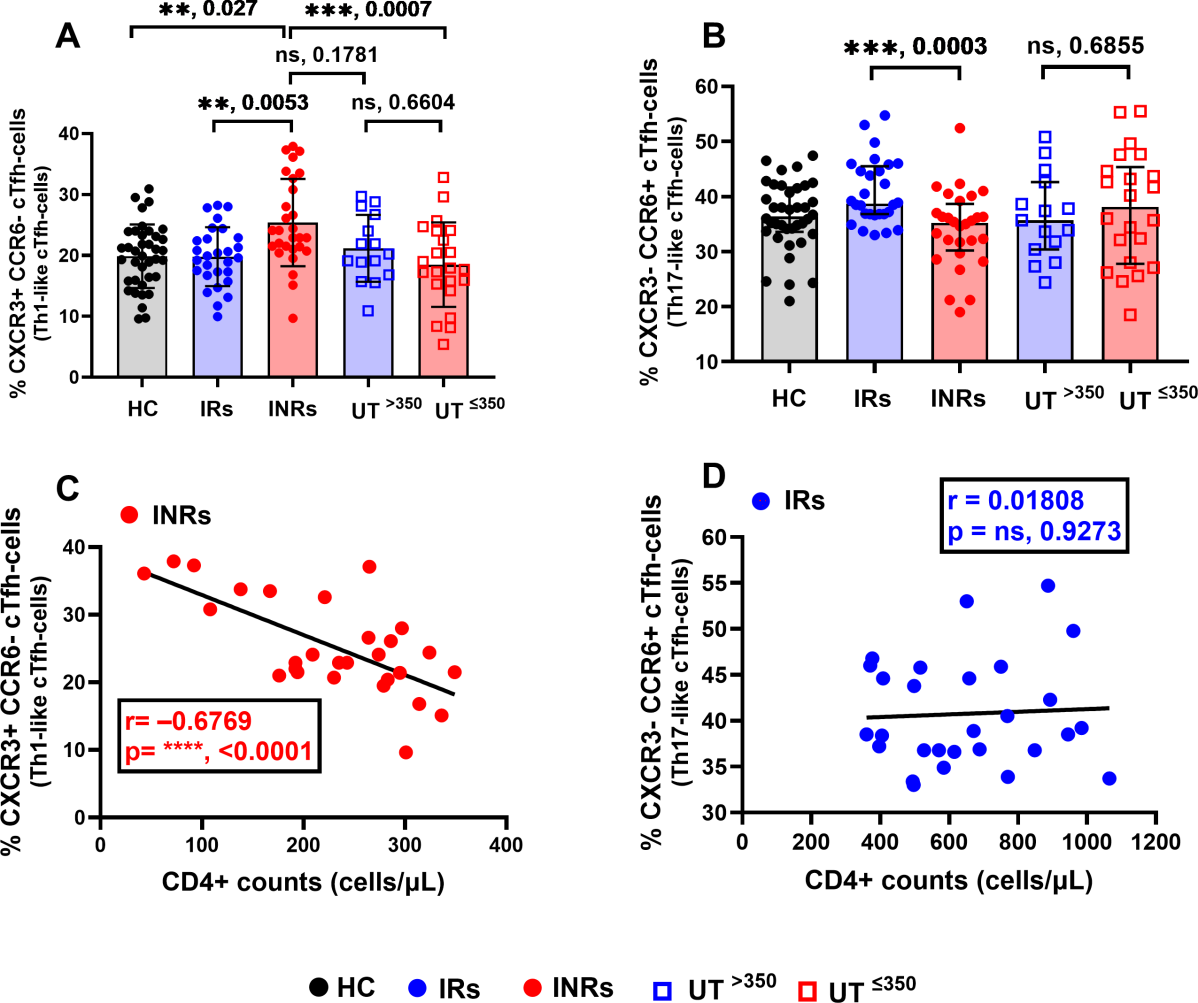
Proportions of CXCR3+ CCR6− Th1-like cTfh cells are associated with poor CD4+ recovery in INRs. Proportions of (A) CXCR3+ CCR6− cTfh cells (Th1-like cTfh cells) and (B) CXCR3− CCR6+ cTfh cells (Th17-like cTfh cells) among the groups. Correlation between CD4+ counts and (C) Th1-like cTfh cells in INRs or (D) Th17-like cTfh cells in IRs. Differences between the two groups were analyzed using the Mann-Whitney test (B). One-way ANOVA was used for multiple group comparisons (A). Pearson (C) or Spearman (D) correlation was used to determine the association between variables (C and D). *P < 0.05, **P < 0.01, ***P < 0.001, ****P < 0.0001, ns: not significant. Each data point represents a single individual. cTfh cells, CXCR5+ CD4+. Black solid circles, HC; blue solid circles, IRs; red solid circles, INRs; blue squares, UT^>350^; and red squares, UT^≤350^.

To assess the link between proportions of helper CD4+ subsets and CD4+ recovery, we quantified total CD4+, cTfh cells (CXCR5+ CD4+), non-cTfh cells (CXCR5− CD4+), and subsequently, quantified their T-helper and helper-like subsets as described in Materials and Methods. The proportions of total CD4+ T cells and non-cTfh cells with T-helper phenotypes (Th1, Th2, and Th17 or non-cTfh1, non-cTfh2, and non-cTfh17) between the groups with high and low CD4+ T-cell counts were not significantly different (see Fig. S3 at https://figshare.com/s/4d69d02499e92beeb30d). However, proportions of CXCR3+ CCR6− cTfh cells (Th1-like cTfh cells—cTfh1) were significantly elevated in INRs compared to HC, IRs, and UT^≤350^ groups (Fig. 5A). Additionally, a strong negative correlation (*r* = –0.6769, *P* < 0.0001) between CXCR3+ CCR6− cTfh cells and CD4+ counts was observed exclusively in INRs (Fig. 5C). These results suggest the skewing of cTfh cells toward Th1-like phenotype, and therefore, CD4+ non-restoration in INRs is probably by cTfh1-mediated depletion. Whereas, in the IRs, proportions of CXCR3− CCR6+ cTfh cells (Th17-like cTfh cells—cTfh17) were significantly increased compared to the HC group and INRs (Fig. 5B), with no correlation (*r* = –0.01801, *P* = 0.9273) with CD4+ counts (Fig. 5D).

## DISCUSSION

The loss of CD4+ during HIV infection is restored by suppressive ART in most people, referred to as IRs. However, despite long-term suppressive ART, a considerable proportion fails to restore their CD4+ counts, referred to as INRs. To delineate the factors associated with impaired immune reconstitution in INRs, this study sought to understand the dynamics of CD4+ subset distribution among HIV-1-infected people, with high or low CD4+ counts, in the presence or absence of ART. We performed a comprehensive analysis of multiple subsets of CD4+ such as naïve, resting, activated (early and late), memory subsets (TCM-TTM and TEM), TEFF, Tregs, cytolytic, cTfh CD4+ T cells, and T-helper and helper-like subsets among the study groups. Studies with such extensive analysis are lacking globally, particularly in India, where HIV-1 subtype C is predominant. This study highlights that the altered proportions of CXCR3+ CCR6− cTfh cells are prominent exclusively in INRs, and their strong association with incomplete CD4+ recovery serves as a hallmark of impaired CD4+ reconstitution.

The study reiterated the fact that delayed ART is one of the causes for impaired CD4+ recovery, with no influence of gender, and low CD4+ groups comprised higher proportions of males probably due to delay in seeking therapy and care (19, 28).

Several studies have highlighted the significance of Tregs in controlling exaggerated immune activation in the context of HIV-1 infection (29). While some studies have shown a negative correlation, others have reported a positive correlation between the frequencies of Tregs and immune activation (29). Therefore, the role of immune activation and Tregs in HIV-1 disease may be either protective or detrimental (29–31). Among HIV-1-infected long-term non-progressors and elite controllers, Treg levels were comparable to healthy controls with increased immune activation. However, among HIV-exposed seronegative commercial sex workers and neonates born to HIV-positive mothers, proportions of Tregs were increased, and levels of T cell activation were decreased, signifying resistance to HIV-1 infection (13, 29).

Notably, similar to other studies (12–14, 32), increased frequencies of both Tregs and activated CD4+ observed in INRs in the present study may suggest a detrimental role (29). A strong negative correlation between CD4+ counts and the proportion of both Treg and activated CD4+ among treated groups suggests their possible association with CD4+ non-restoration. Increased Tregs in the INRs may occur as a result of exaggerated T-cell activation or enhanced thymic output, dendritic cell-mediated conversion and/or persistence, and/or the slow decay of pre-existing Tregs (29, 31). Elevated proportions of activated CD4+ may suggest persistent immune activation probably due to low levels of viral replication in INRs (33). Furthermore, though virologically suppressed, the presence of anti-HIV-1 antibodies (anti-HIV-1-gp41 and anti-HIV-1-gp120) (results not shown) in both the treated groups may imply the existence of residual HIV-1 activity in IRs and INRs. Hence, the higher activated CD4+ in INRs are perhaps sustained from those established pre-ART or stimulated by unknown mechanisms upon initiation of ART, which need further investigation. No difference in CD69+ CD4+ proportions observed between either IRs and INRs or UT^>350^ and UT^≤350^ suggesting no influence on CD4+ counts.

CD57-expressing CD4 T cells are considered proliferation incompetent, cytolytic, and terminally exhausted (senescent). They are shown to produce interferon-gamma (IFN)-γ, granzyme B, and perforin, indicative of cytolytic function. These are reported to display increased levels of spontaneous and activation-induced apoptosis than the CD4+ cells lacking CD57 expression (34, 35). The increased levels of CD57+ CD4+ observed in both the HIV-1-infected low CD4+ groups, irrespective of ART, and a weak negative correlation with CD4+ counts among treated groups suggest their possible role in CD4+ depletion in INRs.

Decreased naïve CD4+ (TN) in HIV-1-infected individuals with low CD4+ counts, with either a decrease (24) or an increase in memory subsets (6, 15, 36) have been reported. The increased proportions of TCM-TTM and a decreased ratio of proportions of TN:TCM-TTM among HIV-1-infected individuals with low CD4+, irrespective of ART, observed in our study, are possibly due to the augmented differentiation of activated CD4+ into memory cells (37, 38). The significantly decreased proportions of TN in the group UT^≤350^ probably suggest depletion and inadequate regeneration of TN (39). The lower trend in TN in INRs, though not significantly reduced, probably suggests ongoing replenishment, depletion, and differentiation of TN into memory CD4+ (4, 40). These observations were alongside significantly reduced proportions of RTEs in the group UT^≤350^, but not in INRs, suggesting thymic deficiency (inadequate regeneration of CD4+) in the untreated condition and its restoration upon ART (4, 8, 23), ruling out thymic insufficiency in INRs. This is also evidenced by the restoration and comparable proportions of cTfh cells in both the treated groups, which were significantly depleted in both the untreated groups. The weak positive and negative correlation observed among TN and TCM-TTM with CD4+ counts, respectively, probably suggests their partial association with CD4+ recovery. Lower CD31+ memory cells in INRs suggest higher differentiation of RTEs into CD31− (loss of CD31 expression) memory CD4+ or increased peripheral proliferation (26, 27). Factors responsible for higher levels of TN differentiation into memory CD4+ require further research.

cTfh cells are recognized for their support to help B cells in enhanced antibody production (41, 42). Studies have shown that helper-like cTfh subsets such as CXCR3+ (cTfh1) and CXCR3− (cTfh2 and cTfh17) cTfh cells correlate with enhanced antigen-specific antibody production, either against HIV, hepatitis C virus (HCV), during influenza vaccination (43), or autoimmune conditions (particularly cTfh17 cells) (41, 43–46). These studies indicate that cTfh subset polarization is context-dependent, such as virus type, infection state, or immunogen (43). Among helper and helper-like CD4+ subsets, polarized proportions of Th1-like cTfh cells (CXCR3+ CCR6− CXCR5+ CD4+, cTfh1 cells) in INRs imply that Th1-like cTfh cells may induce antigen-specific antibody response. Though the target-specific antibodies in INRs were not identified in our study, they are possibly auto-reactive anti-CD4+ T-cell antibodies (11, 47–49), strongly suggesting cTfh1-cell-mediated CD4+ depletion. A strong negative correlation between proportions of cTfh1 cells and CD4+ counts exclusively in INRs suggests a major contribution of elevated cTfh1 cells in CD4+ non-restoration. Our study and other studies highlight the need for additional investigations to identify epitope-specific anti-CD4+ T-cell auto-antibodies or approaches to inhibit cTfh1-cell responses that result in CD4+ depletion. Either of which would prevent CD4+ T-cell loss and help restoration. On the contrary, the predominant proportions of Th17-like cTfh cell (CXCR3− CCR6+ CXCR5+ CD4+, cTfh17 cells) in IRs may indicate autoimmune antibody responses (41, 46), rather than their association with CD4+ restoration as reported (50), which was not observed in our study.

Taken together, our results demonstrate that exclusively elevated proportions of CXCR3+ CCR6− cTfh cells and their strong negative correlation with CD4+ counts in INRs suggest an association with CD4+ depletion. Elevated proportions of Tregs and activated CD4+ in INRs and their strong negative correlation with CD4+ counts among treated groups indicate an association with CD4+ non-recovery. Higher cytolytic CD4+ and TCM-TTM showed a weak association with CD4+ non-recovery. Increased TCM-TTM in INRs is due to increased peripheral proliferation or differentiation of RTEs into memory cells. There was no evidence of thymic deficiency in INRs. CXCR3− CCR6+ cTfh cells in IRs may suggest their role in autoimmunity, rather than CD4+ recovery.

This study has a few limitations. A longitudinal study design could have made the analysis more robust, enabling early prediction of INRs. The mechanism underlying altered CD4+ subsets, host genetic factors affecting immune responses, the lifestyle of study subjects, viral aspects, and their impact on CD4+ non-restoration in INRs have not been investigated due to limited resources.

### Conclusions

We postulate that in INRs, the cTfh cells are polarized by an unknown mechanism toward cTfh1 (CXCR5+ CXCR3+ CCR6− CD4+) upon initiation of ART, which may contribute to CD4+ non-restoration. The cTfh1-mediated immune dysregulation may result in altered Tregs and late-activated CD4+ in INRs. The comprehensive assessment of multiple subsets of CD4+ aided the simultaneous identification of specific altered patterns that can serve as biomarkers for impaired CD4+ reconstitution. The study findings can drive future research to develop cTfh1-targeted interventions to facilitate CD4+ recovery. This, in turn, can contribute to lowering the increased risk of developing AIDS and non-AIDS complications in INRs.

## MATERIALS AND METHODS

### Study design and population

The study was primarily cross-sectional and conducted from 2022 to 2024. The HIV-1-infected individuals who sought treatment at the ART Center, Kempegowda Institute of Medical Sciences & Research Center (KIMS & RC), Bangalore, India, were recruited. The study groups included healthy controls (uninfected), apparently healthy and negative for HIV-1/2, hepatitis B virus (HBV), and hepatitis C virus (HCV) infections (HC, *n* = 38); HIV-1-infected individuals on long-term ART for 2–14 years (treated) with CD4+ counts either >350 cells/µL (IRs, *n* = 28) or ≤350 cells/µL (INRs, *n* = 28); ART-naïve HIV-1-infected individuals (untreated) with CD4+ counts >350 cells/µL (UT^>350^, *n* = 15) and those with ≤350 cells/µL (UT^≤350^, *n* = 22). These groups are addressed as HC, IRs, INRs, UT^>350^, and UT^≤350^, respectively. Consenting male, female, and transgender individuals aged above 18 years were recruited.

### HIV diagnosis, plasma viral load, and CD4+ counts

CD4+ counts and viral load details of HIV-1-positive individuals at recruitment, before and after initiation of ART were obtained from their medical records. Plasma samples of apparently healthy subjects were used to exclude HIV-1/HIV-2, HCV, and HBV-infected individuals by using rapid card tests (Diagnostic Enterprises, India) such as HIV Tri-Dot kit, HCV Tri-Dot kit, and Hepacard, respectively. Their CD4 counts were measured in whole blood using a CD4 easy count kit in the Sysmex Partec CyFlow flow cytometer (Sysmex Partec GmbH, Germany) by trained personnel.

### Identification of subsets of CD4+ between the groups

CD4+ subsets were identified and quantified by their expression of the following surface markers: Tregs (CD4+ CD25+/hi CD127lo/−) (16), resting (CD4+ CD25− CD69− HLA-DR−), TN (CD4+ CD27+ CD45RO−), TEM (CD4+ CD27− CD45RO+), TCM-TTM (CD4+ CD27+ CD45RO+), TEFF (CD4+ CD27− CD45RO−) (51), cytotoxic (CD4+ CD57+) (34), CD69+ (early activation marker) CD4+, activated (CD4+ CD38+ HLA-DR+; late activation markers) (21, 22, 24), RTEs (CD4+ CD31+ CD27+ CD45RO−), CD31+ memory subsets: CD31+ TEM (CD4+ CD31+ CD27− CD45RO+), CD31+ TCM-TTM (CD4+ CD31+ CD27+ CD45RO+), and CD31+ TEFF (CD4+ CD31+ CD27− CD45RO−) (26). Total CD4+ were classified into two major subsets based on the expression or lack of CXCR5 chemokine receptor: (i) cTfh cells (CXCR5+ CD4+) and (ii) non-cTfh cells (CXCR5− CD4+). Subsequently, these subsets were further subtyped into T-helper and helper-like subsets based on the expression of CXCR3 and CCR6, such as Th1 (CD4+ CXCR3+ CCR6−), Th2 (CD4+ CXCR3− CCR6−), and Th17 (CD4+ CXCR3− CCR6+) under total CD4+, cTfh1 (CD4+ CXCR5+ CXCR3+ CCR6−), cTfh2 (CD4+ CXCR5+ CXCR3− CCR6−), and cTfh17 (CD4+ CXCR5+ CXCR3− CCR6+) under cTfh cells, and non-cTfh1 (CD4+ CXCR5− CXCR3+ CCR6−), non-cTfh2 (CD4+ CXCR5− CXCR3− CCR6−), and non-cTfh17 (CD4+ CXCR5− CXCR3− CCR6+) under non-cTfh cells (43, 52). A representative figure showing the gating strategy for the identification and quantification of all CD4+ subsets is shown in Fig. S1 at https://figshare.com/s/4d69d02499e92beeb30d.

### Immunophenotyping of peripheral blood mononuclear cells (PBMCs) by flow cytometry

PBMCs isolated from blood by Ficoll density gradient centrifugation were stained with Live/Dead Fixable Green and with respective antibody panels. The antibodies procured from either BD Biosciences or BioLegend included the following: anti-CD3 (UCHT1 or SK7), anti-CD4 (SK3), anti-CD8 (SK1), anti-CD25 (M-A251), anti-CD69 (FN50), anti-HLA-DR (G46-6), anti-CD127 (hIL-7R-M21 or A019D5), anti-CD38 (HIT2 or HB7), anti-CD31 or anti-PECAM-1 (WM59), anti-CD27 (L128), anti-CXCR5 (RF8B2 or J252D4), anti-CD45RO (UCHL1), anti-CD57 (NK-1), CXCR3 (1C6), and CCR6 (G034E3). Stained cells were acquired using a FACSVerse (BD Bioscience) flow cytometer. Data were analyzed using Flowjo version 10.0.8 (BD Bioscience).

### Statistical analysis

A Fisher’s test was used to compare categorical data. Data that followed the Gaussian distribution were analyzed using parametric tests; otherwise, non-parametric tests were used. The unpaired *t*-test or non-parametric Mann-Whitney *U* test was used to compare two independent groups. For multiple-group comparisons, one-way ANOVA or non-parametric Kruskal-Wallis with Dunn’s multiple comparison tests were used. Pearson or non-parametric Spearman correlation tests were used to determine the association between variables. A *P* value of ˂0.05 was considered statistically significant. Statistical analysis was performed using GraphPad Prism 10 (version 10.2.2 [397]).
